# Hierarchical Clustering Analysis for Positioning Two Intrusion Events at Different Locations Using Dual Mach-Zehnder Interferometers

**DOI:** 10.3390/s25165074

**Published:** 2025-08-15

**Authors:** Ting-Wang Chen, Likarn Wang

**Affiliations:** Institute of Photonics Technologies, National Tsing Hua University, Hsinchu 300, Taiwan; penguin8591@gmail.com

**Keywords:** intrusion detection, dual Mach-Zehnder interferometer, hierarchical clustering analysis, two simultaneously-occurring intrusion events

## Abstract

Hierarchical clustering analysis is applied to the positioning of two simultaneously-occurring intrusion events in the case of a dual Mach-Zehnder interferometer used for intrusion detection. To simulate the two intrusion events, the sensing fibers of the dual Mach-Zehnder interferometer are heavily knocked at two different positions simultaneously. Then the clockwise (CW) and counter-clockwise (CCW) signals are loaded into a personal computer through a data acquisition module, and analyzed by Fourier transform method for determination of the time delay between the two signals. Hierarchical clustering analysis is then employed twice for dividing the data points in a feature space into several clusters according to the conditions required. To locate the two intrusions, the first clustering analysis is performed on the data points formed by signals detected in a 10 ms time period, with the centroid of the largest cluster being the location of a single intrusion event. Then, 100 pairs of CW and CCW signals detected sequentially are analyzed to give 100 locations. These 100 locations and their CP values (each standing for a ratio of a given spectral amplitude to the summation of the spectral amplitudes over the frequency band of 2500 to 5000 Hz) constitute 100 data points in a feature space for the second hierarchical clustering analysis, which then determines the respective locations of the two intrusion events. In the test of a 1036 m long fiber perimeter, we demonstrated an accuracy to within 21.55 m.

## 1. Introduction

There are a variety of fiber-optic sensors for perimeter intrusion detection, such as fiber Bragg grating sensors [1,2], Rayleigh backscattering based time-domain reflectometric sensors [3,4,5,6], and optical interferometric sensors, the last of which covers the use of techniques using Michelson interferometers [7,8], Sagnac interferometers [9,10], and Mach-Zehnder interferometers [11,12]. Techniques with merged types of interferometer such as those reported in the works of Refs. [13,14,15,16] also supported good location ability in disturbance detection.

Recently, dual Mach-Zehnder interferometers (DMZIs) have been studied for accurate location of the intrusion event occurring along sensing fibers. An experimental test demonstrated an average locating error of 390 m by using an 18.46 km long sensing interferometer [17]. With a polarization feedback-loop control used to eliminate the effect of polarization induced fading (PIF), a locating error of 160 m for a 112 km long DMZI was obtained [18]. Then, to mitigate the effect of PIF, a chaotic particle swarm optimization-based algorithm was used, achieving a locating error of ±20 m over a 2.25 km sensing cable [19], which was claimed to be one order of magnitude lower than that obtained by using a conventional interference visibility-based polarization control algorithm. To eliminate the influence of Rayleigh backscattering on the counter-clockwise (CCW) and clockwise (CW) signals in the DMZI system, a wavelength-division multiplexing technique was employed, achieving an accuracy to within 52.5 m in the case of a 61 km sensing length, which was much better than that obtained for a traditional DMZI [20]. Faraday rotating mirrors were used to eliminate the effect of PIF, leading to a locating error of ±25 m in the experiment using a 100-km sensing distance [21]. Most of the present DMZIs, like those mentioned above, used a cross correlation algorithm to determine the time delay between CCW and CW signals acquired, which is equivalent to the location of intrusion.

A Fourier spectral analysis method was presented to determine the location of an intrusion event without using the conventional cross-correlation method for a modified DMZI intrusion detection system with a locating error of <26 m for a 250 m (or 1036 m) long detection range [22]. The effect of PIF was reported not to appear in the long-term test for such a modified DMZI system. Later, a hierarchical clustering analysis method for this DMZI system was applied to determine the location of an intrusion event with maximum mean locating errors of 11.55 m [23].

However, all present DMZIs can locate only one intrusion event at a time. Researchers have achieved some progress toward simultaneously locating multiple events along the sensing fibers. Sun et al. proposed a way of locating multiple vibrations of different frequencies at different positions in their experiment with a ring-type Mach-Zehnder interferometer, although they addressed only the detection of multiple events of different frequencies at the same position [24]. On the other hand, Zhang et al. proposed an algorithm for locating two disturbances occurring simultaneously at different positions, but without experimental confirmation [25]. To address the issue of locating multiple intrusion events that simultaneously occur at different positions, this paper provides more concrete experimental location results for two simultaneous events. The rest of the paper is organized as follows. Section 2 outlines the principle of the proposed optical method for detecting two simultaneous intrusion events. In Section 3, experimental results are presented by using the hierarchical analysis method to find the absolute error in the positioning of two intrusion events. Section 4 concludes this work.

## 2. Outline of the Presented Measurement System

### 2.1. Detected Signals

**Figure 1:**
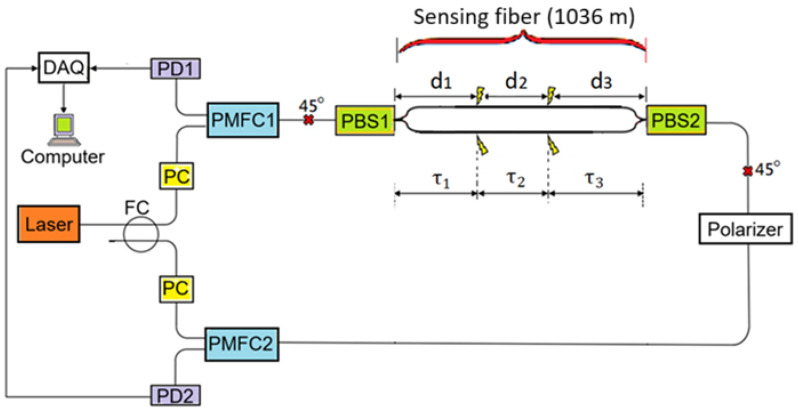
DMZI system for positioning two different intrusion events occurring at different locations. FC: 3 dB fiber coupler; PD1 and PD2: photodetector; PC: polarization controller; DAQ: data acquisition card; PMFC1 and PMFC2: polarization maintaining fiber coupler; PBS1 and PBS2: polarization beam splitter. The symbol × denotes 45 degree splicing between the principal axes of two polarization maintaining fibers.

We use our previous modified DMZI presented in [22] for intrusion positioning in this study. Figure 1 shows the DMZI with two polarization beam splitters/combiners (PBS1 and PBS2) used for an MZI structure. Two polarization-maintaining fiber couplers (PMFC1 and PMFC2) are also used to couple two waves into one path (or split one wave into two) for only one linearly polarized wave, e.g., the x polarized wave (i.e., they only operate for the x-polarized wave.). Each PC (polarization controller) is adjusted to obtain an output wave in a state approaching an x-polarized wave. There are two sites with 45 degree splicing between two principal axes of the spliced polarization-maintaining fibers (see cross marks in the figure). One site is between PMFC1 and PBS1, and the other site is between PBS2 and the Polarizer (which is an inline fiber polarizer with a polarization-maintaining fiber at its two ends).

A laser light is first split into two paths through a fiber coupler (FC). One of the split lights propagates through a PC, PMFC1, the MZI, the inline fiber polarizer, PMFC2, and is then detected by a photodetector (i.e., PD2). This light is referred to as CW light because it propagates clockwise in a loop. After the clockwise light passes through PMFC1 and the 45 degree splicing site, there are x-polarized and y-polarized light along the polarization maintaining fiber at the input end of PBS1. After propagating through PBS1, only x-polarized light and y-polarized light would propagate in the upper and the lower arms of the MZI, respectively. The two orthogonally-polarized lights then combine via PBS2, and interfere through the inline fiber polarizer, which delivers the interfered light along its polarization-maintaining fiber output end. The light then propagates along the fiber and reaches PMFC2 and then PD2. A CCW light is defined for the other split light, which propagates though a PC, PMFC2, the inline fiber polarizer, the MZI, PMFC1, and then is detected by PD1. A more detailed description of the operation of the modified DMZIs can be found in [22].

Two intrusions are supposed to occur simultaneously at two different positions such that the fibers are disturbed at positions z = d_1_ and z = d_1_ + d_2_, respectively, from PBS1, as shown in Figure 1. The time it takes the light to travel over the distance of d_1_, d_2_ and d_3_ is denoted by τ_1_, τ_2_ and τ_3_, respectively.

Suppose the phases incurred by the disturbances at z = d_1_ are $\Phi_{X1}(t)$ (upper arm) and $\Phi_{Y1}(t)$ (lower arm), while the disturbances at z = d_1_ + d_2_ are $\Phi_{X2}(t)$ (upper arm) and $\Phi_{Y2}(t)$ (lower arm). Then the detected optical powers by PD1 and PD2 can be expressed, respectively, as

(1) $$I_{PD1}\left(t\right)=I_{1}[1+\cos\left(\Phi_{1}\left(t-(\tau +\tau_{2}+\tau_{3}\right)\right)+\Phi_{2}\left.\left(t-(\tau +\tau_{3}\right)\right))]$$

and

(2) $$I_{PD2}\left(t\right)=I_{2}[1+\cos\left(\Phi_{1}\left(t-\tau_{1}\right)+\Phi_{2}\left(t-{(\tau }_{1}+\tau_{2}\right))\right)]$$

where I_1_ and I_2_ are both constants, and $\Phi_{1}(t)$ and $\Phi_{2}(t)$ are $\Phi_{X1}\left(t\right)-\Phi_{Y1}(t)$ and $\Phi_{X2}\left(t\right)-\Phi_{Y2}(t)$, respectively. Then, we define $\Phi_{12}\left(t\right)=\Phi_{1}\left(t\right)+\Phi_{2}(t-\tau_{2})$ and $\Phi_{21}\left(t\right)=\Phi_{2}\left(t\right)+\Phi_{1}(t-\tau_{2})$. Equations (1) and (2) can thus be rewritten as

(3) $$I_{PD1}\left(t\right)=I_{1}[1+cos(\Phi_{21}\left(t-\tau -\tau_{3}\right))]$$

and

(4) $$I_{PD2}\left(t\right)=I_{2}[1+cos(\Phi_{12}\left(t-\tau_{1}\right))],$$

respectively.

There exists a range of $\tau_{2}$ such that $\Phi_{12}\left(t\right)$ equals $\Phi_{21}\left(t\right)$. Therefore, Equations (3) and (4) can be re-expressed as

(5) $$I_{PD1}\left(t\right)\equiv I_{1}[1+cos(\Phi_{12}\left(t-\tau -\tau_{3}\right))]$$

(6) $$I_{PD2}(t)\equiv I_{2}[1+cos(\Phi_{12}\left(t-\tau_{1}\right))]$$

We further explain the limitation of the range of $\tau_{2}$ for $\Phi_{12}\left(t\right)$ being equal to $\Phi_{21}\left(t\right)$. In fact, we need ω $\tau_{2}$ to be small enough for exp(ω $\tau_{2}$) to approximately be equal to 1. In the study, the range of ω is from 2π·2000 to 2π·5000, and thus 2π·5000$\tau_{2}$ should be small enough. This corresponds to the case that the separation between the two intrusion events should be smaller than 6366 m. The smaller the separation, the truer $\Phi_{12}\left(t\right)$ equals $\Phi_{21}\left(t\right)$. In this study, the maximum separation would be 1036 m, which naturally makes $\Phi_{12}\left(t\right)$ approximately equal to $\Phi_{21}\left(t\right)$. Because of this limitation of the separation, the maximum length of the DMZIs should also be smaller than 6366 m.

From Equations (5) and (6), the time delay between $I_{PD1}\left(t\right)$ and $I_{PD2}\left(t\right)$ is ${(\tau }_{1}+\tau_{2}+\tau_{3}+\tau_{3})-\tau_{1}=\tau_{2}+2\tau_{3}$ when there are two simultaneous intrusion events occurring at z = d_1_ and z = d_1_ + d_2_. If $\tau_{2}\;=\;0$, the time delay becomes $2\tau_{3}$, and this case corresponds to an intrusion event occurring at z = L − v·$\tau_{3}$, or at a position of v·$\tau_{3}$ away from PBS2, where L is the total length of the sensing fiber and v the light speed in the fiber. However, the time delay induced by the two simultaneous intrusion events is definitely mistaken for a case of only one intrusion event occurring at a position of v·($\tau_{2}+2\tau_{3}$)/2.

**Figure 2:**
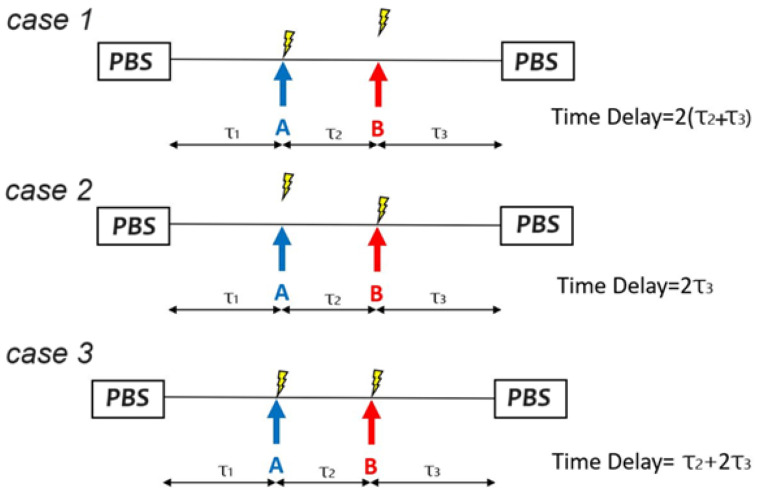
Practical situations when two intrusion events occur simultaneously at positions A and B. Case 1: only one intrusion occurs at position A. Case 2: only one intrusion occurs at position B. Case 3: intrusions occur simultaneously at positions A and B. PBS: polarization beam splitter used for the MZI.

In the study, the detected signals I_PD1_ and I_PD2_ are acquired by a data acquisition module (DAQ) every 10 ms at a sample rate of 2 Msamples/s, and each acquired signal waveform has a time interval of 10 ms. In such a short time interval, the sensing fiber might be disturbed in three ways as shown in Figure 2, where the fiber is disturbed only at position A (case 1), only at position B (case 2), and simultaneously at positions A and B (case 3). When the fiber is disturbed at only one position, as in case 1 or case 2 of Figure 2, the intrusion location can be determined as usual by finding the time delay between the two detected signals I_PD1_ and I_PD2_, i.e., the CCW and CW signals. On the other hand, when the fiber is disturbed simultaneously at positions A and B, as in case 3 of Figure 2, there exists a time delay of $\tau_{2}+2\tau_{3}$ between the two detected signals I_PD1_ and I_PD2_. Note that this time delay is equal to the average of the two time delays for cases 1 and 2, but is incorrect for determining the intrusion position. In summary, only the time delays in cases 1 and 2 are correct in determining the locations of disturbances, while the time delay in case 3 is not.

As aforementioned, the detected signals I_PD1_ and I_PD2_ are acquired every 10 ms and thus 100 pairs of detected signals would be acquired continuously within 1 s. For each pair, one time delay approaching 2 (τ_2_ + τ_3_), 2τ_3_ or $\tau_{2}+2\tau_{3}$ can be found by hierarchical clustering analysis.

### 2.2. Hierarchical Clustering Analysis for Positioning

One time delay value will be obtained by using a hierarchical analysis, as we did in [23], and this time delay corresponds to a position which is defined by the distance from PBS2. The distance is expressed by position number, which is proportional to a real distance. The position number is defined to be a real distance from PBS2 (in meters) divided by 50. Here, 50 is the spatial resolution in meters resulting from the sampling rate of the DAQ used in the experiments. In this study, a unit value of position number represents a real distance of 50 m as in the work of [23]. In the first-step hierarchical analysis, feature coordinates such as (x, y), where x represents the position number and y the CP value, are used for each pair of detected signals. This x coordinate approaches one of the two intrusion positions, i.e., v·(τ_2_ + τ_3_) and v·$\tau_{3}$, or approaches v·($\tau_{2}+2\tau_{3}$)/2, which are all expressed in terms of position number. By using 100 consecutive detected signals, we have 100 such kinds of feature coordinate. Then, by using these 100 feature coordinates as data points for the feature space in a hierarchical analysis, we seek the centroids of three largest clusters. Here, the x coordinates of the centroids represent the position numbers that could correspond to the intrusion positions for the three cases in Figure 2. The largest and the smallest position numbers of the three thus obtained then represent the two true intrusion positions as revealed by cases 1 and 2, respectively, and the position number between the largest and the smallest is false, which correspond to the time delay $\tau_{2}+2\tau_{3}$ as revealed in case 3 of Figure 2. One hundred pairs of consecutive detected signals correspond to 100 data points in the feature space. We chose 100 data points because they correspond to detected signals acquired within 1 s time duration, and such time duration is considered to be proper here. If fewer data points are taken, the position of the centroid of each cluster in the second-step hierarchical analysis might be less accurate. In an opposite case with more data points, the positioning accuracy might be relatively high. However, the latter case refers to a longer positioning time required.

In this study, the 1036 m long sensing fiber of the DMZIs is continuously knocked at two positions to simulate simultaneous intrusions. The two intrusions occur simultaneously at two of the three positions that are at 1 m, 249 m and 643 m away from PBS2. These three positions correspond to position numbers of 0.02 (1 m), 4.98 (249 m), and 12.86 (643 m), respectively. That is, we consider three situations, i.e., intrusions occur simultaneously at positions 0.02 and 4.98 for situation 1, 0.02 and 12.86 for situation 2, and 4.98 and 12.86 for situation 3. When the sensing fiber is knocked in each situation, a series of I_PD1_ and I_PD2_ are obtained.

## 3. Experimental Results

### 3.1. Hierarchical Clustering Results for Intrusions at 0.02 and 4.98

**Figure 3:**
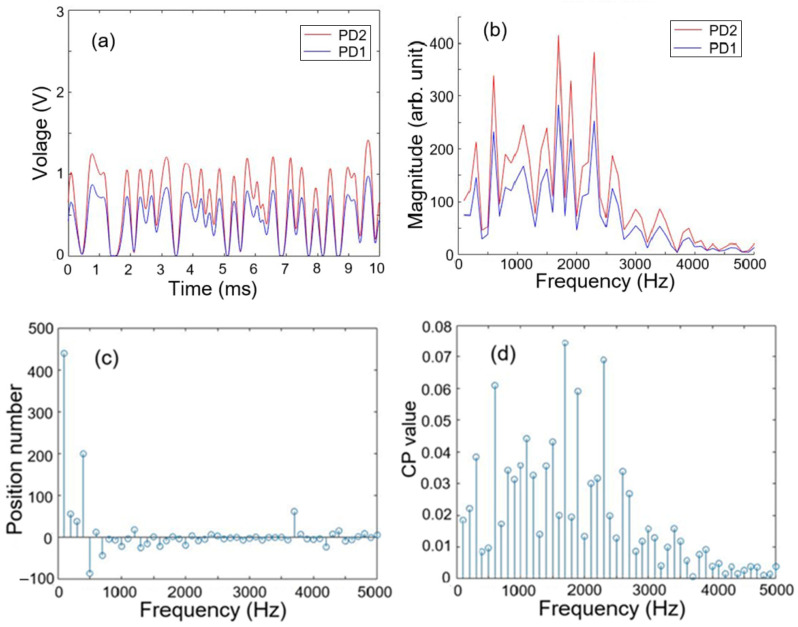
Detected signal waveforms of I_PD1_ and I_PD2_ (**a**) and their signal spectra (**b**). Spectrum of position number (**c**) and spectrum of CP value (**d**).

Figure 3a,b shows the detected signal waveforms and their Fourier spectral amplitudes for the situation in which intrusions occur simultaneously at positions 0.02 and 4.98. Since the detected CW and CCW signals have a time delay between them, as revealed in the three cases of Figure 2, there is a phase shift spectrum that shows the dependence of time delay on frequency, and henceforth the dependence of position number on frequency, as shown in Figure 3c. On the other hand, from Figure 3b, we can see a spectrum of CP value, which is defined as a ratio of a given spectral amplitude to the summation of the spectral amplitudes over the frequency band of 2500 to 5000 Hz. The spectrum of CP value is shown in Figure 3d.

**Figure 4:**
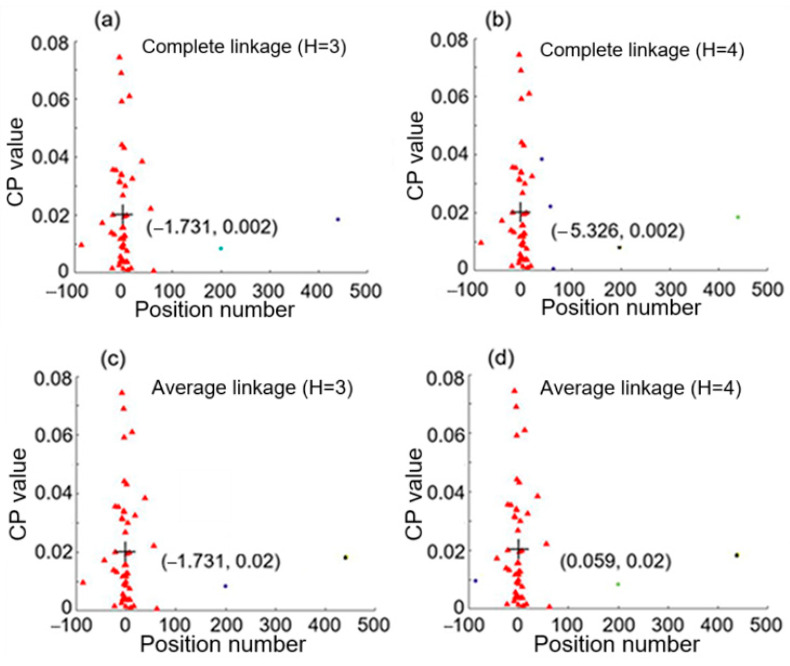
Hierarchical clustering results for knocking simultaneously at positions 0.02 and 4.98. (**a**,**b**) are obtained by using complete linkage with cluster number H equal, respectively, to 3 and 4, while (**c**,**d**) are obtained by using average linkage with cluster number H equal, respectively, to 3 and 4.

From Figure 3c,d, a position number at a given frequency reflects a CP value at that frequency. These two figures reveal that there is a relation between position number and CP value according to the spectral dependence. Therefore, they are used as a data point in the 2D feature space for hierarchical clustering analysis, and we will have 50 data points in the space, each corresponding to a specific frequency from 100 Hz to 5000 Hz at an interval of 100 Hz. Figure 4 shows the clustering results with complete linkage and average linkage used. Here the cluster numbers (i.e., the number of groups) H are chosen to be 3 and 4. Data points of different clusters are represented by different symbols, which have different shapes or colors for different clusters. The centroid of the largest group (or cluster) is marked by + and its coordinates are denoted. We can see that average linkage with H = 4 used in the clustering analysis revealed in Figure 4d gives the largest cluster having a centroid with the coordinates (0.059, 0.02), where the x coordinate 0.059 is close to 0.02 and represents only one intrusion event occurring as shown case 2 of Figure 2. In this case, the detected signals shown in Figure 3a yields only one single intrusion event occurring at position 0.059, which denotes a discrepancy of 0.039, i.e., 1.95 m in real distance, with respect to the real intrusion position 0.02. It is noted that this accurate result was obtained by using average linkage with H = 4, and that complete linkage or H = 3 cannot lead to such a result.

**Figure 5:**
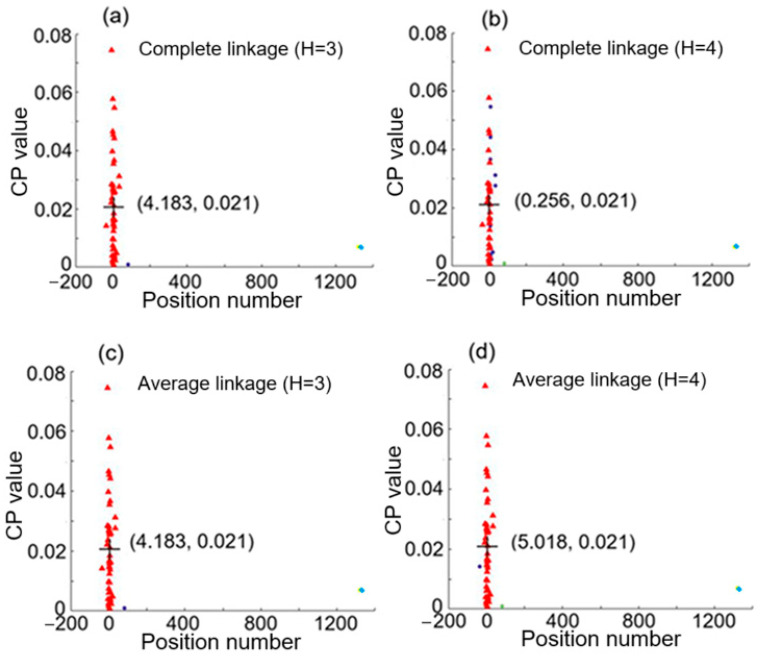
Hierarchical clustering results for knocking simultaneously at positions 0.02 and 4.98. (**a**,**b**) are obtained by using complete linkage with cluster number H equal, respectively, to 3 and 4, while (**c**,**d**) are obtained by using average linkage with cluster number H equal, respectively, to 3 and 4.

We then chose another pair of I_PD1_ and I_PD2_, and performed the hierarchical clustering analysis. Figure 5 shows the clustering results with complete linkage (H = 3 and 4) and average linkage (H = 3 and 4). Then, we found that average linkage using H = 4 gives centroid coordinates of (5.018, 0.021) for the largest cluster. The x coordinate 5.018 is quite close to 4.98, meaning that this pair of detected signals represents a single intrusion at position 5.016 with an error of 0.036 (i.e., an error of 1.8 m in real distance), as illustrated by case 1 of Figure 2.

**Figure 6:**
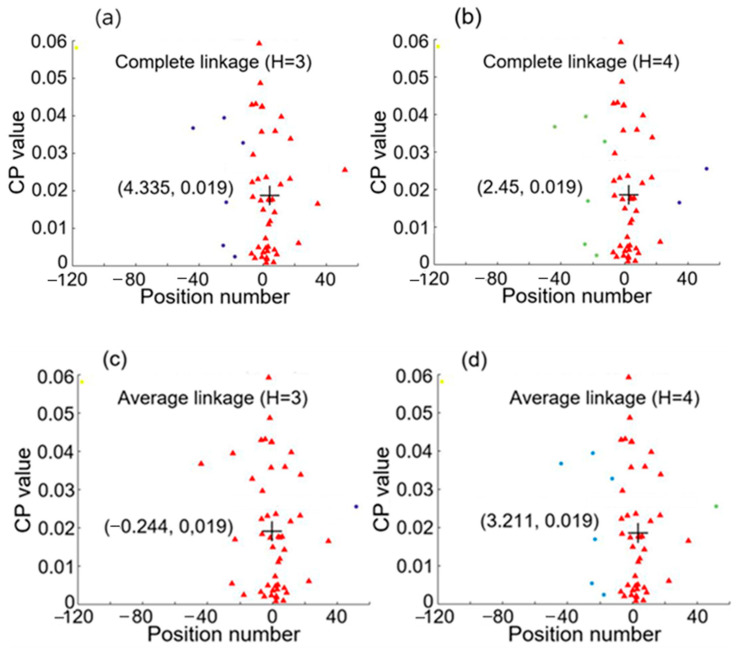
Hierarchical clustering results for knocking simultaneously at positions 0.02 and 4.98. (**a**,**b**) are obtained by using complete linkage with cluster number H equal, respectively, to 3 and 4, while (**c**,**d**) are obtained by using average linkage with cluster number H equal, respectively, to 3 and 4.

Then, again we chose another pair of I_PD1_ and I_PD2_, and performed the hierarchical clustering analysis. In this example, there is no position number approaching 0.02 and 4.98, and Figure 6b gives a centroid with its x coordinate approaching the average of 0.02 and 4.98, as is indicated by case 3 of Figure 2.

We also performed the first-step hierarchical clustering analysis when the sensing fibers were knocked simultaneously at two positions of 0.02 and 12.86 for situation 2, and 4.98 and 12.86 for situation 3. It was found that cases 1, 2 and 3 of Figure 2 existed for knocking at the two positions in situations 2 and 3. Then, we carried out an investigation such that the only partial band from 2000 Hz to 5000 Hz in the spectra of position number and CP value was considered and used to establish the 2D feature space with only 31 data points. This was carried out so that the intrusion position could be determined either to be more close to one of the two knocking positions, as revealed by cases 1 and 2 of Figure 2, or to be more close to the middle value of the two knocking positions, as indicated in case 3 of Figure 2, in the hierarchical clustering analysis, compared with the case when the full spectra of position number and CP value were used.

Next, we used 100 pairs of I_PD1_ and I_PD2,_ consecutively acquired for every 1 s, to define the two intrusion positions. Each pair of detected signals is analyzed using complete linkage and average linkage with cluster number H = 3, 4, and 5, and considering the frequency band from 2000 Hz to 5000 Hz in the spectra of position number and CP value. In the hierarchical clustering analysis with each algorithm model, we then obtain 100 position numbers, which correspond to the three cases of Figure 2, and their associated CP values. Then, these 100 position numbers and CP values will be used for the second hierarchical clustering analysis with complete linkage and average linkage, using the cluster number H2 = 3, 4, 5, 6, 7, 8. In each of the second hierarchical clustering analysis, we will usually obtain the three largest clusters, and the x coordinates of their centroids correspond to the three cases of Figure 2. That is, we will have three position numbers, a smallest position number, a largest position number and an average value of the two position numbers. From interpretation of Figure 2, the smallest and the largest position number obtained are the two real intrusion positions, approximately, with little error. From this analysis, we can see which algorithm model (i.e., complete linkage or average linkage with an associated cluster number) achieves the best results.

**Figure 7:**
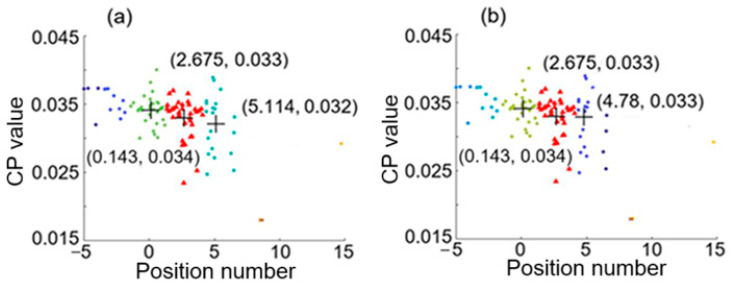
Feature space with three largest clusters formed by using the first hierarchical clustering analysis with average linkage of H = 3 and the second hierarchical clustering analysis with (**a**) average linkage of H2 = 7 and (**b**) average linkage of H2 = 8, for the case of two intrusion positions at 0.02 and 4.98.

Figure 7 shows a feature space with the three largest clusters formed by using the first hierarchical clustering analysis with average linkage of H = 3 and the second hierarchical clustering analysis with (a) average linkage of H2 = 7 and (b) average linkage of H2 = 8, for the case of two intrusion positions at 0.02 and 4.98. From the smallest and largest values of the x coordinates of the three centroids marked by the symbol +, we can see that these results give 0.143 and 5.114 (or 4.78) for the two intrusion positions, and these estimated values are in a good agreement with the two real intrusion positions at 0.02 and 4.98.

**Figure 8:**
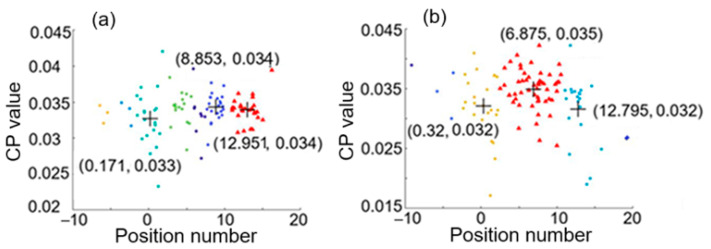
Feature space with the three largest clusters formed by using (**a**) the first hierarchical clustering analysis with average linkage of H = 3 and the second hierarchical clustering analysis with average linkage of H2 = 7 and (**b**) the first hierarchical clustering analysis with average linkage of H = 4 and the second hierarchical clustering analysis with average linkage of H2 = 6, for the case of two intrusion positions at 0.02 and 12.86.

Figure 8 shows a feature space with the three largest clusters formed by using (a) the first hierarchical clustering analysis with average linkage of H = 3 and the second hierarchical clustering analysis with average linkage of H2 = 7, and by using (b) the first hierarchical clustering analysis with average linkage of H = 4 and the second hierarchical clustering analysis with average linkage of H2 = 6, for the case of two intrusion positions at 0.02 and 12.86. The two positions estimated are 0.171 and 12.951, respectively, as indicated by Figure 8a. The discrepancies between the real positions and the estimated positions are both small (smaller than 7.6 m in real distance).

**Figure 9:**
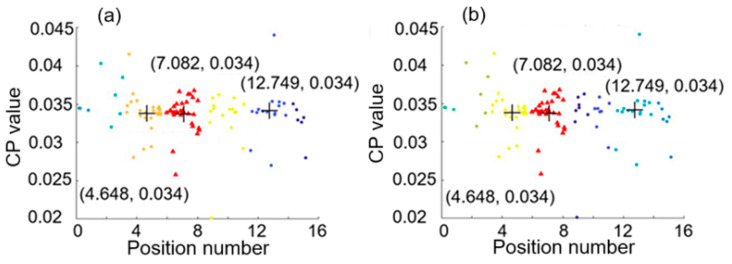
Feature space with the three largest clusters formed by using the first hierarchical clustering analysis with average linkage of H = 3 and the second hierarchical clustering analysis with (**a**) average linkage of H2 = 7 and (**b**) average linkage of H2 = 8, for the case of two intrusion positions at 4.98 and 12.86.

For the case of two intrusion positions at 4.98 and 12.86, the feature spaces with the three largest clusters formed by using the first hierarchical clustering analysis with average linkage of H = 3 and the second hierarchical clustering analysis with average linkage of H2 = 7 and average linkage of H2 = 8 are shown in Figure 9a and Figure 9b, respectively. The estimated positions are 4.684 and 12.749 from either Figure 9a or Figure 9b, which are close to the real positions 4.98 and 12.86, respectively.

### 3.2. Absolute Error in Positioning of Two Intrusion Events

Note that, in this study, each intrusion event is assumed to last for one second, and 100 pairs of detected signals are acquired for two hierarchical clustering analyses. In the first hierarchical clustering analysis, each pair of detected signals was analyzed using complete linkage and average linkage with cluster number H = 3, 4, and 5 while, in the second hierarchical clustering analysis, 100 data points were analyzed using complete linkage and average linkage with the cluster number H2 = 3, 4, 5, 6, 7, 8. We knocked the sensing fibers simultaneously at two different positions to obtain 18 sets of detected signals with each set lasting for 1 s. That is, we disturbed the fibers 18 times and that represented 18 intrusion events simultaneously occurring at two positions. It was found that the first hierarchical clustering analysis with average linkage used for H = 3 and H = 4 could achieve the best positioning performance, together with the second hierarchical clustering analysis with average linkage for H2 = 7. Below, we use absolute error to define the magnitude of positioning discrepancy between the estimated intrusion position and the real intrusion position for each intrusion event. The absolute errors were computed by using the first hierarchical clustering analysis with average linkage for H = 3 and H = 4 and using the second hierarchical clustering analysis with average linkage for H2 = 7, and are shown in the following figures.

**Figure 10:**
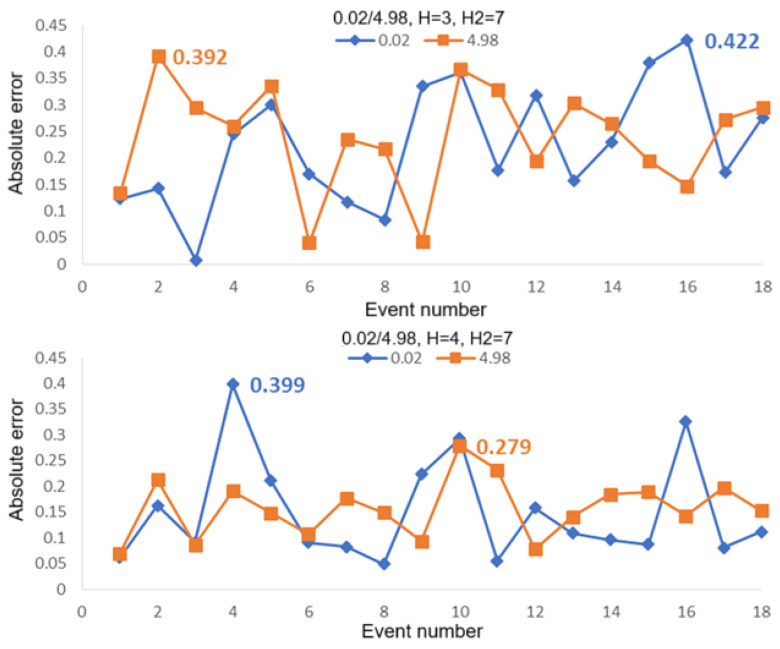
Absolute errors computed for 18 intrusion events by using the first hierarchical clustering analysis with average linkage for H = 3 (**upper**) and H = 4 (**lower**). The second hierarchical clustering analysis was used with average linkage for H2 = 7 in the two figures for the case of intrusion positions 0.02 and 4.98.

**Figure 11:**
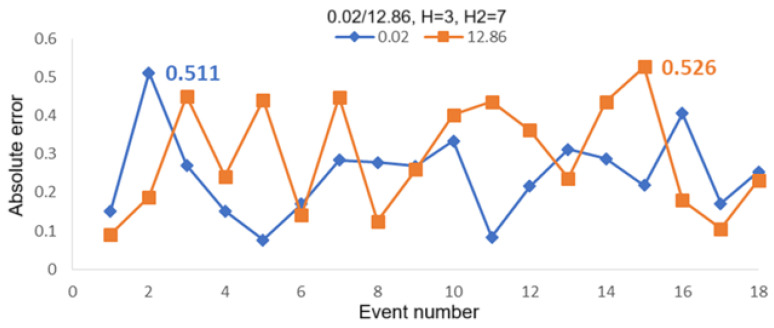

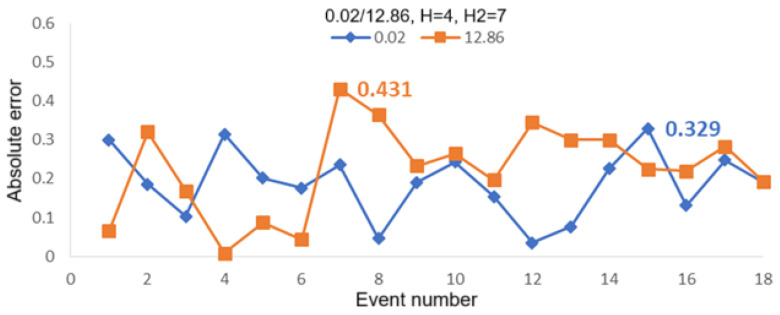
Absolute errors computed for 18 intrusion events by using the first hierarchical clustering analysis with average linkage for H = 3 (**upper**) and H = 4 (**lower**). The second hierarchical clustering analysis was used with average linkage for H2 = 7 in the two figures for the case of intrusion positions 0.02 and 12.86.

**Figure 12:**
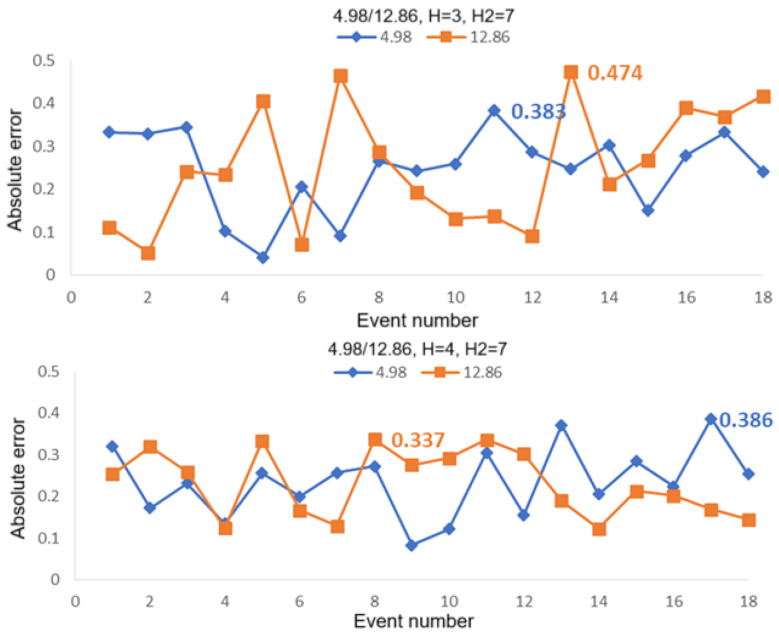
Absolute errors computed for 18 intrusion events by using the first hierarchical clustering analysis with average linkage for H = 3 (**upper**) and H = 4 (**lower**). The second hierarchical clustering analysis was used with average linkage for H2 = 7 in the two figures for the case of intrusion positions 4.98 and 12.86.

Figure 10 shows the computed absolute errors for the case of two intrusion positions of 0.02 and 4.98 for 18 intrusion events. Average linkage with H2 = 7 was used in the second hierarchical clustering analysis, while average linkage with H = 3 (upper figure) and H = 4 (lower figure) were used in the first hierarchical clustering analysis. It can be seen from this set of figures that the maximum absolute error for H = 3 is 0.422, corresponding to 21.1 m in real distance, and is 0.399 for H = 4, corresponding to 19.95 m in real distance. Figure 11 shows the computed absolute errors for the case of two intrusion positions of 0.02 and 12.86 for 18 intrusion events. The maximum absolute errors are 0.526 (corresponding to 26.3 m in real distance for H = 3) and 0.431 (corresponding to 21.55 m in real distance for H = 4). Figure 12 shows the computed absolute errors for the case of two intrusion positions of 4.98 and 12.86 for 18 intrusion events. The maximum absolute error is 0.474 for H = 3, corresponding to 23.7 m in real distance, while it is 0.386 (corresponding to 19.3 m in real distance) for H = 4. Here, the maximum absolute errors are all obtained by using average linkage in the first and the second hierarchical clustering analyses. It can be seen that, if we choose H = 4 in the first hierarchical clustering analysis, the maximum errors would be smaller compared with those for H = 3.

## 4. Conclusions and Discussion

A clustering analysis method has been used for determining the positions of two intrusion events simultaneously occurring at two different positions. The basic principle of this means of locating relies on the fact that there are three cases that can occur for the two simultaneous intrusions. By using a first hierarchical clustering analysis for every pair of CW and CCW detected signals that are acquired within 10 ms, we can obtain three results, and there are three positions found. Besides the two intrusion positions, we can find a position that is between these two. By denoting an intrusion event within 1 s, we then detect 100 pairs of CW and CCW signals by applying a second hierarchical clustering analysis to choose the correct two positions for the intrusion events. In a test of 18 sets of simultaneous intrusion events, we have the largest position error of 21.55 m if an average linkage with a cluster number of 4 in the first hierarchical clustering analysis and an average linkage with a cluster number of 7 in the second hierarchical clustering analysis are used.

To maintain a good interference visibility at the output end of the MZI, the x-polarized light at the upper arm and that at the lower arm must have equal power. Once good visibility is achieved, the shapes of the CCW and CW signals are the same, except for a time delay that corresponds to the disturbance location. To maintain an equal power between the two polarized lights over short lengths, the fiber cable that contains the sensing fibers cannot be bent randomly to avoid coupling between x and y polarizations in the fiber. However, it is inevitable that such coupling may occur over a long fiber that extends to regions with a varying temperature distribution.

To finding the centers of clustering in each step, we need to calculate the spectra of position number and CP values, as indicated by Figure 3c,d. Then we find the centroid of the cluster in the first step for processing each of the 100 pairs of signals, and the three centroids of the clusters would be found in the second step of the hierarchical clustering analysis. The computational cost required for implementing a real-time system with the two-step hierarchical clustering algorithm and intrusion positioning depends on software handling and memory required. Present computer science technology can handle this kind of work. Therefore, deploying a prompt positioning algorithm based on the two-step hierarchical clustering analysis is feasible.

The concept of quantum-enhanced sensing for a nonlinear interferometer was brought up to possibly improve the phase estimation accuracy of the interferometer [26] and could minimize the positioning error for DMZIs. Practical work for applying such a quantum-enhanced sensing theory to positioning of the intrusion events in DMZI remains as an interesting issue.

## Data Availability

The data presented in this study are available in this article.
